# Mapping the future: Two decades of insights into preoperative frailty assessment in elderly patients

**DOI:** 10.1016/j.dialog.2026.100352

**Published:** 2026-09-25

**Authors:** Yuling Ni, Naiyang Tang, Xi Xu

**Affiliations:** a Anesthesiology, Qidong Traditional Chinese Medicine Hospital, Qidong 226200, China; b Support Department, Unit 31635 of PLA, Guilin 541000, China

**Keywords:** Preoperative frailty assessment, Elderly patients, Sarcopenia, Malnutrition, Depression, Bibliometric analysis

## Abstract

**Background:**

The assessment of preoperative frailty (PFA) has become an essential instrument for forecasting postoperative outcomes in elderly patients (EP). However, there is a notable absence of comprehensive bibliometric analyses that chart global research trends in this area. This study systematically investigates the intellectual landscape, key research areas, and evolving paradigms in PFA research for EP from 2005 to 2025.

**Methods:**

Publications from 2005 to 2025 were retrieved from the Web of Science Core Collection using a comprehensive search strategy that integrated terms associated with EP and PFA. Analytical tools VOSviewer 1.6.19 and CiteSpace 6.4.R1 were utilized to examine annual publication trends, country and institutional contributions, journal and authorship patterns, co-citation networks, and keyword emergence.

**Results:**

Among 2108 English-language full-text articles and reviews, the annual publication output increased quadratically, reaching its peak in 2024. The United States led in research productivity (805 papers; 22,402 citations), followed by Europe. Harvard Medical School was the most prolific institution, contributing 59 papers. *JAMA Surgery* garnered the highest number of citations (2399) and had the highest impact factor (14.9). The keywords “sarcopenia,” “depression,” and “malnutrition” demonstrated the most significant recent citation bursts, indicating emerging research hotspots.

**Conclusions:**

PFA in EP is rapidly expanding, with a predominant contribution from North American and European research. Future priorities should focus on incorporating sarcopenia, depression, and malnutrition into standardized preoperative protocols to facilitate personalized risk stratification and the development of tailored prehabilitation strategies.

## Highlights

• First bibliometric analysis of preoperative frailty in elderly patients.
• Global publications on preoperative frailty assessment grew exponentially.
• USA and Europe dominated the research output and international collaborations.
• Sarcopenia, depression, and malnutrition emerged as key research hotspots.

## 1 Introduction

Frailty, a geriatric syndrome characterized by multisystem physiological decline, heightens susceptibility of patients to stressors and predisposes them to adverse clinical outcomes [1]. Evidence consistently demonstrates that frailty is significantly associated with increased 30 and 180-day postoperative mortality across diverse surgical specialties [2]. In cardiac surgery, preoperative frailty assessment (PFA) has emerged as a robust predictor of postoperative disability or mortality, outperforming conventional risk stratification tools [3]. Notably, prehabilitation has shown promise in ameliorating frailty status and potentially reducing postoperative complications in this vulnerable population [4].

PFA in elderly patients (EP), particularly those with severe comorbidities, serves as a critical tool for clinicians to predict postoperative outcomes and tailor individualized treatment strategies [5]. Among older adults undergoing aortic valve replacement, those with higher preoperative frailty levels exhibit significantly reduced likelihood of functional recovery within one-year post-surgery [6]. Comprehensive Geriatric Assessment (CGA) and Frailty Index (FI) have emerged as validated instruments for identifying high-risk patients, not only predicting postoperative complications but also guiding personalized care plans to enhance quality of life after surgery [7]. Plent of original articles and reviews have been published on PFA for EP each year, suggesting that prehabilitation will advance rapidly in the next few years. Currently, there are no visual analyses of publications related to PFA for EP.

In a wide range of disciplines, bibliometrics are widely used to evaluate research trends and hotspots [8], [9]. While Chen et al. [10] provided a comprehensive bibliometric analysis of perioperative care for elderly patients, highlighting the rising focus on postoperative cognitive dysfunction and organ protection, their study lacked dedicated evaluation of PFA for EP. Using bibliometric software such as VOSviewer and CiteSpace, we conducted a systematic analysis and visualization of PFA for EP publications over the past 20 years. This study aims to address the critical knowledge gap regarding the lack of standardized, globally recognized clinical pathways for PFA. By mapping the intellectual landscape and identifying emerging micro-level clinical targets, this study seeks to provide an evidence-based foundation for future multicenter trials and the development of tailored prehabilitation policies.

## 2 Methods

### 2.1 Data source

The Web of Science Core Collection (WOSCC) now comprises over 12,400 high-quality, authoritative academic journals.The WoSCC database was utilized to analyze bibliometric data, as bibliometric mapping tools like CiteSpace 6.4.R1 and VOSviewer 1.6.19 require specific WoSCC data formats for network creation.In order to execute its calculations, bibliometric software necessitates data information from this database.The advanced search was configured as: “TS = ((elderly OR aged OR geriatric* OR ‘old patient*’) AND (preoperative OR ‘pre-operative’ OR ‘before surgery’ OR ‘prior to surgery’) AND (frail* OR fragility OR ‘frailty index’ OR ‘frailty phenotype’ OR ‘frailty assessment’ OR ‘frailty screening’) AND (evaluat* OR assess* OR screen* OR predict* OR identif*))”. Papers and reviews were selected and downloaded on September 11, 2025, covering the period from September 11, 2005, to September 11, 2025. An excerpt of 2108 articles was exported as plain text. The analysis was limited to peer-reviewed articles and review articles that were published in English, as these document types contain all the necessary bibliometric metadata. As a result of inconsistent citation patterns and incomplete metadata that could potentially distort network analysis, Proceedings paper, early access, editorial material, meeting abstract, book chapter, letter, retracted publication, and correction were excluded (Fig. 1).

**Fig. 1:**
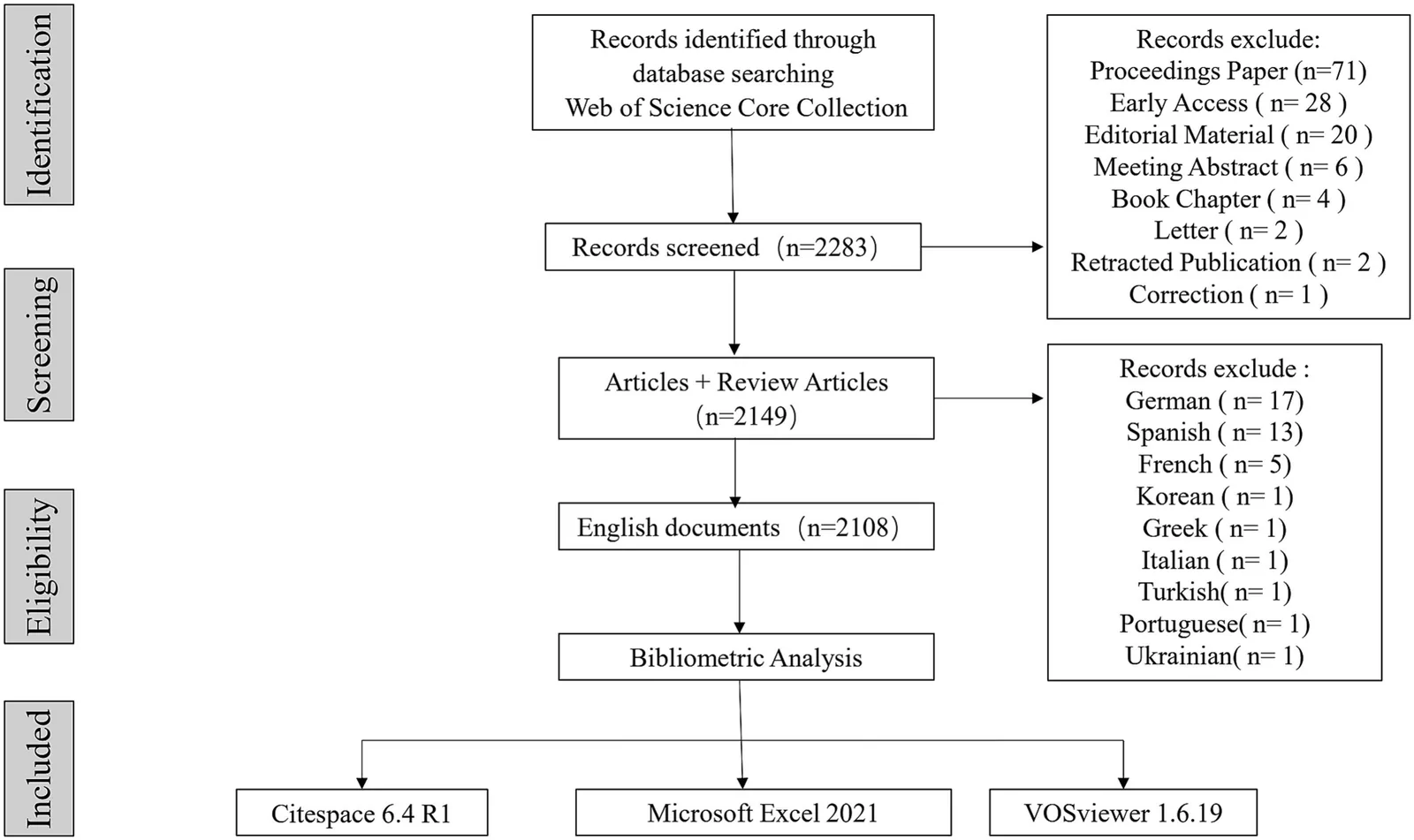
Flow chart of the literature search.

### 2.2 Data analysis

Various bibliometric software tools are currently available, including CiteSpace 6.4.R1 and VOSviewer 1.6.19. CiteSpace's capability to analyze and visualize networks, identify clusters, autonomously assign nomenclature, implement macro theory, overlay various graphs, and highlight pivotal transitions contributed to its selection. VOSviewer was chosen for visual analysis and knowledge mapping due to its distinctive time-overlapping map feature, which is absent in other analytical software. A document statistics chart for state institutions, an annual development chart, and additional data tables were created in Microsoft Excel 2021.

## 3 Results

### 3.1 Annual publications trend

The yearly publication count is a crucial metric that reflects the rate of knowledge progression and demonstrates trends within the field. Our bibliometric analysis encompassed 2108 publications from 2005 to 2025.The yearly publication output exhibited a consistent rise from 2012 to 2022, culminating in a peak in 2024 (Fig. 2). A polynomial curve model was employed to elucidate this trend: y = 0.8846 × ^2^–1.4217×-6.6123 (R^2^ = 0.894), emphasizing the statistical significance of the identified pattern. The results underscore the growing scholarly emphasis on PFA in EP.

**Fig. 2:**
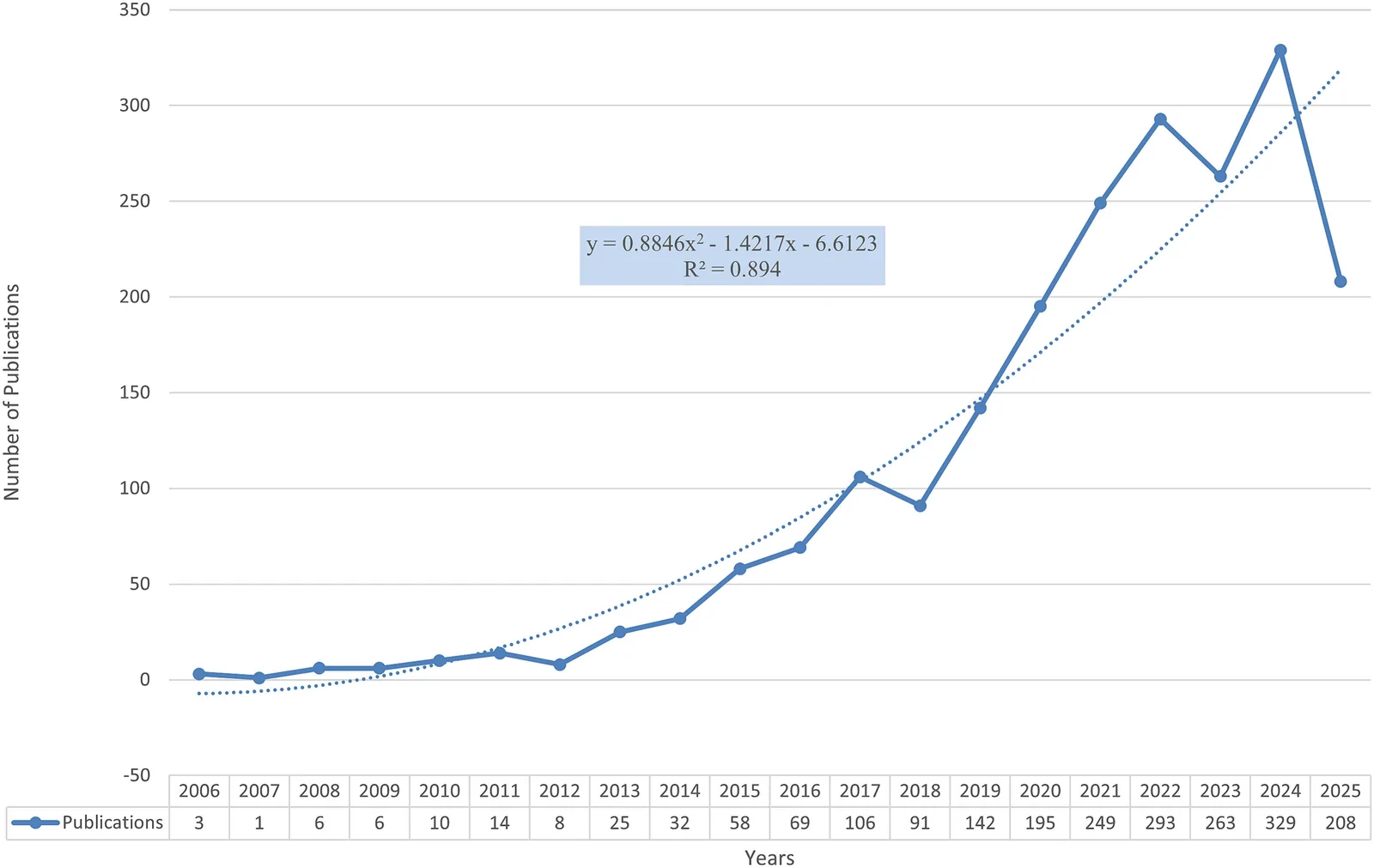
Annual number of publications from 2006 to 2025.

### 3.2 Countries or regions

Fig. 3A depicts the ten top-producing nations in this domain, determined by the efforts of scientists. The USA is characterized by a considerable volume of publications (*n* = 805) and the highest citation count (*n* = 22,402), indicating its prominent scientific output and dominance in PFA of EP. Europe was recognized as a significant contributor, with representation from five countries. Canada ranks fifth in published articles (*n* = 155) and second in citation counts (*n* = 5693), indicating its substantial influence in this domain.

**Fig. 3:**
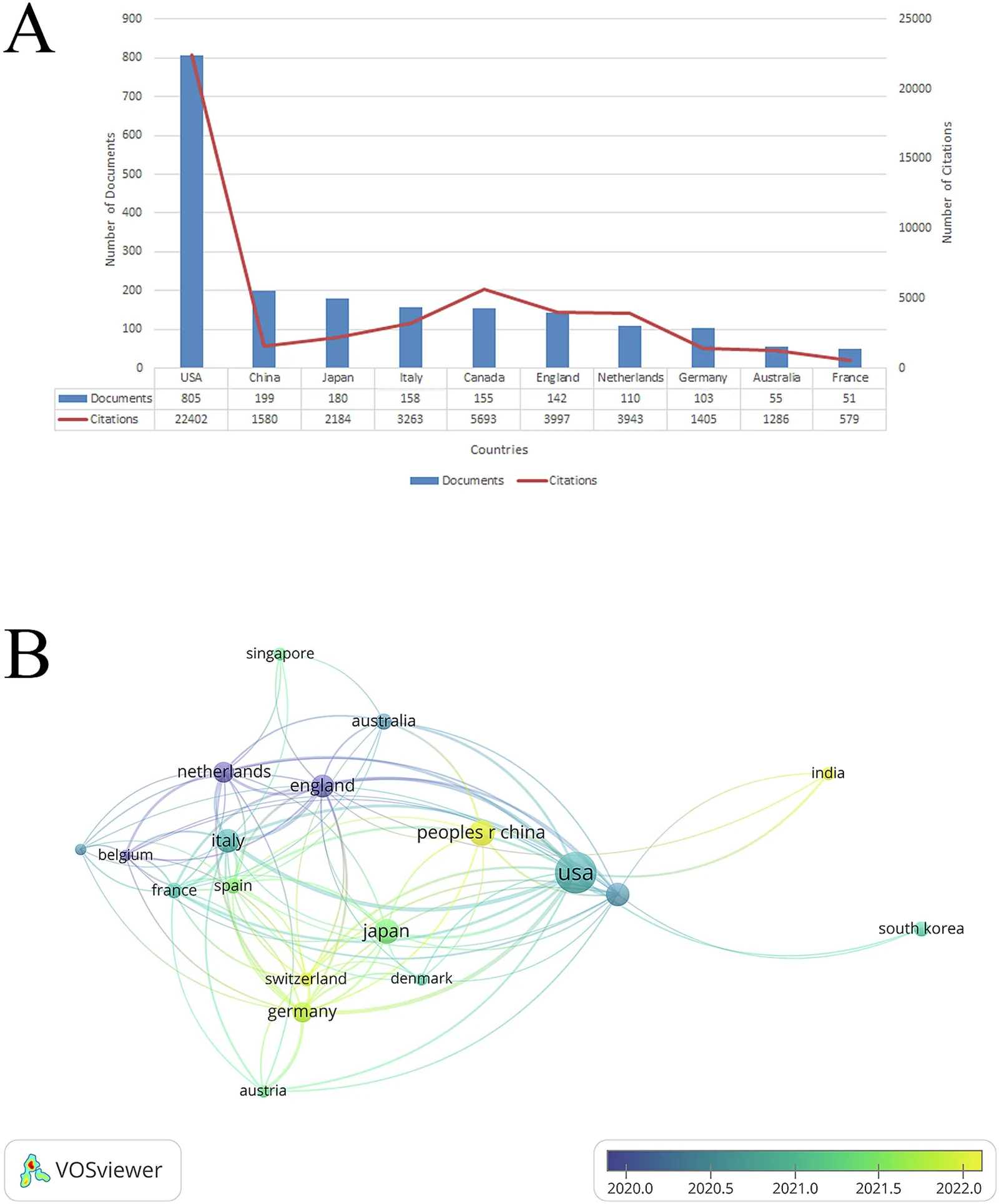
Analysis of countries or regions on research of preoperative frailty assessment for elderly patients. (A) Documents and citations of the top 10 most productive countries or regions; (B) Time-overlapping network.

Fig. 3B depicts the strong collaboration among nations that have published works related to PFA of EP. The USA, China, Japan, and Italy were the nations that engaged in the most collaboration with other countries or regions, aligning with their submission of the highest number of articles. Moreover, England is among the pioneering nations to have undertaken research in this sector, with a significant body of literature emerging in this field in 2020. Moreover, the emergence of Chinese publications in 2022 indicated a recent and rapid expansion of PFA in EP research within China.

### 3.3 Institutes

Fig. 4A demonstrates that a thorough analysis of the top ten prolific institutions in PFA of EP indicates a significant dominance of organizations from the United States and Canada. Specifically, six of these esteemed institutions were situated in the USA, three in Canada, and one in the Netherlands. In the competitive landscape, Harvard Medical School in the USA is the most prolific institution, generating a substantial number of publications (*n* = 59). The University of Groningen in the Netherlands has concurrently been acknowledged for publishing the most frequently cited organization (*n* = 2156).

**Fig. 4:**
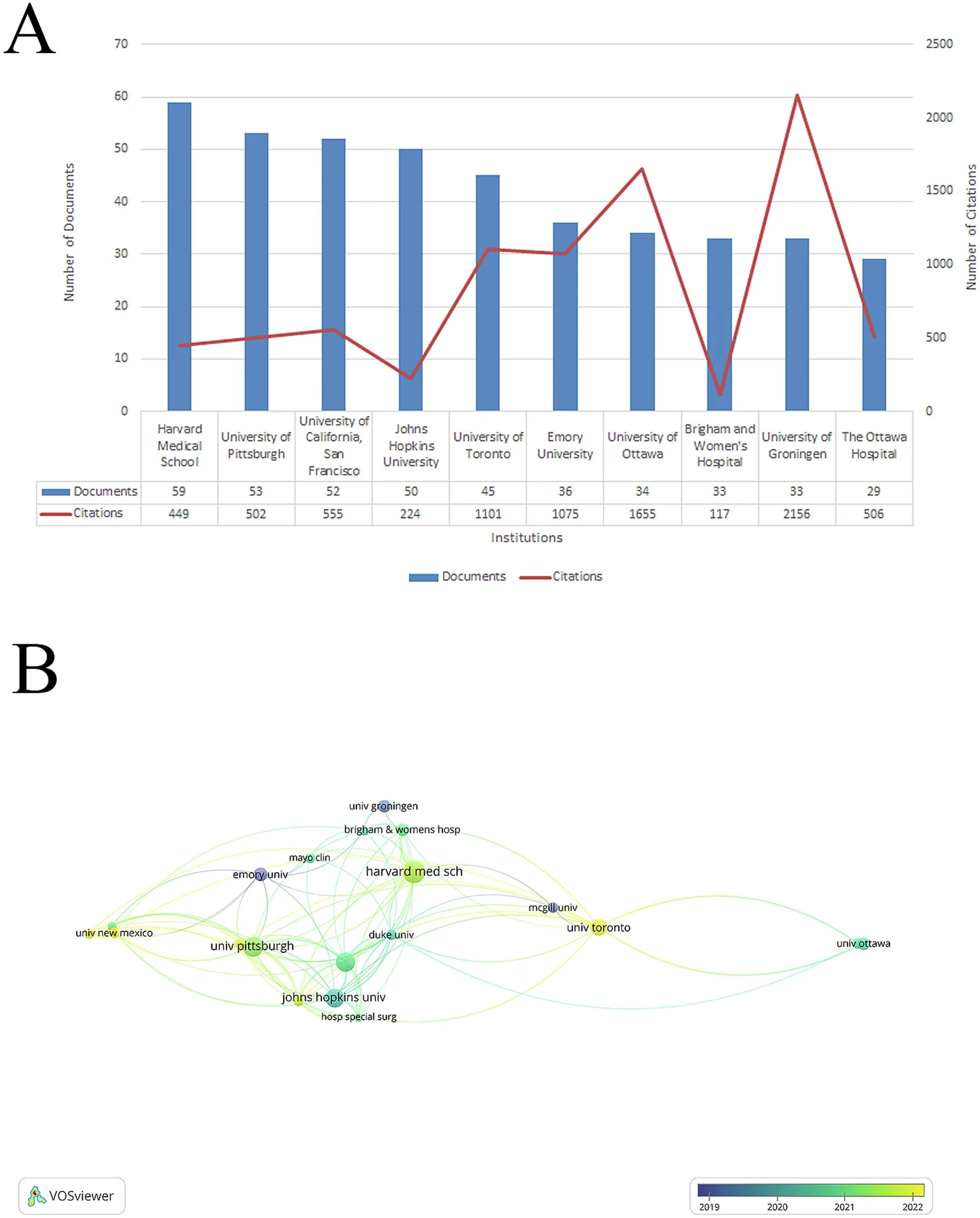
Analysis of institutions on research of preoperative frailty assessment for elderly patients. (A) Documents and citations of the top 10 most productive institutions; (B) Time-overlapping network.

Successful large-scale research is facilitated by the collaboration of multiple institutions. Fig. 4B illustrates that institutional collaborations predominantly centered on institutions in the United States and Canada. Harvard Medical School has partnered with numerous esteemed scientific institutions in the research of PFA of EP over the last ten years. Large yellow nodes signify the University of Toronto and the University of New Mexico, reflecting their designation as emerging research institutions. In contrast, the extent of cooperation between Asian institutions and those in other countries seems to be limited.

### 3.4 Journals

The notable contributions of nations, organizations, and individuals were recorded in academic journals, leading to an examination of their publication and dissemination. Table 1 illustrates that among the top ten relevant journals for PFA of EP, eight are American journals and two are from England. The four primary journal publishers include Elsevier, and two journals are published by Lippincott Williams & Wilkins. The *Journal of Surgical Research* (*n* = 37) and *BMC Geriatrics* (*n* = 37) were the leading journals regarding publication volume.

**Table 1:** The top 10 journals related to research of preoperative frailty assessment for elderly patients.

Ranks	Journal	Documents	Citations	Country	Publisher	IF(2024)	JCR
1	Journal of Surgical Research	37	1436	USA	Elsevier	1.7	Q2
2	BMC Geriatrics	37	333	England	BioMed Central Ltd	3.8	Q1
3	European Journal of Surgical Oncology	35	729	England	Elsevier	2.9	Q1
4	Journal of Clinical Medicine	35	153	USA	MDPI	2.9	Q1
5	Geriatric Orthopaedic Surgery & Rehabilitation	27	329	USA	SAGE Publications	1.6	Q2
6	Anesthesia and Analgesia	26	1205	USA	Lippincott Williams & Wilkins	3.8	Q1
7	Annals of Surgery	26	1894	USA	Lippincott Williams & Wilkins	6.4	Q1
8	Journal of Geriatric Oncology	26	459	USA	Elsevier	2.7	Q3
9	World Neurosurgery	25	326	USA	Elsevier	2.1	Q2
10	JAMA Surgery	22	2399	USA	American Medical Association	14.9	Q1

*JAMA Surgery* was the most cited journal with 2399 citations, followed by *Annals of Surgery* with 1894 citations and another instance of *Annals of Surgery* with 1436 citations. In 2024, the impact factors of these journals ranged from 1.6 to 14.9.Aside from *JAMA Surgery*, the leading journal in this field, the remaining journals had an impact factor below 7.The classification indicated that Q1 accounted for 60% and Q2 for 30%, as established by the Journal Citation Reports (JCR) partition analysis.

### 3.5 Authors

To determine the leading experts in PFA for EP over the past twenty years, authors were ranked based on their publication count. Table 2 presents the ten authors with the highest citation counts in the domain of PFA in EP. Bowers Christian A, Schmidt Meic H., and McIsaac Daniel I. ranked as the top three authors, with publication counts of 35, 24, and 23 articles, respectively. Of the authors, 80% were from the United States, whereas 20% of the top 10 authors were from Canada. Three of the ten most prolific authors are affiliated with the University of New Mexico, and authors with high productivity are linked to institutions that produce a significant number of publications. Arya Shipra and Hall Daniel E. retained the top two positions in citation count, with 1426 and 1288 citations, respectively, in the analysis of the most influential studies on PFA in EP.

**Table 2:** The top 10 authors related to research of preoperative frailty assessment for elderly patients.

Ranks	Authors	Documents	Citations	Institutes	Countries
1	Bowers, Christian A	35	377	University of New Mexico	USA
2	Schmidt, Meic H.	24	302	University of New Mexico	USA
3	McIsaac, Daniel I.	23	1024	University of Ottawa	Canada
4	Kazim, Syed Faraz	23	291	University of New Mexico	USA
5	Hall, Daniel E.	18	1288	University of Pittsburgh	USA
6	Arya, Shipra	15	1426	Emory University	USA
7	Lafage, Virginie	15	531	Hospital for Special Surgery	USA
8	Shahrokni, Armin	15	307	Memorial Sloan Kettering Cancer Center	USA
9	Afilalo, Jonathan	13	552	McGill University	Canada
10	Sciubba, Daniel M.	13	418	Long Island Jewish Medical Center	USA

### 3.6 Documents and co-cited references

Table 3 presents an overview of the 10 highest cited articles in the discipline of PFA for EP [11], [12], [13], [14], [15], [16], [17], [18], [19], [20]. The 10 articles received citations ranging from 331 to 1570, including titles such as “Comprehensive geriatric assessment can predict complications in elderly patients after elective surgery for colorectal cancer: A prospective observational cohort study “ [20] and “ Frailty as a predictor of surgical outcomes in older patients “ [11]. Seventy percent of these papers were obtained from the Journal of JCR Q1, whereas 30 % originated from JCR Q2. Eighty of the ten research investigations investigated the relationship between frailty and surgical results in elderly individuals, whereas the other two focused on sarcopenia.

**Table 3:** The top 10 cited documents related to research of preoperative frailty assessment for elderly patients.

Ranks	Documents	Citations	Title	Journal	IF(2024)	JCR
1	Makary (2010) [11]	1570	Frailty as a predictor of surgical outcomes in older patients	Journal of the American College of Surgeons	3.4	Q1
2	Velanovich (2013) [12]	647	Accumulating deficits model of frailty and postoperative mortality and morbidity: its application to a national database	Journal of Surgical Research	1.7	Q2
3	Ethun (2017) [13]	452	Frailty and cancer: Implications for oncology surgery, medical oncology, and radiation oncology	CA: A Cancer Journal for Clinicians	232.4	Q1
4	Peng (2012) [14]	449	Impact of sarcopenia on outcomes following resection of pancreatic adenocarcinoma	Journal of Gastrointestinal Surgery	1.7	Q2
5	Reisinger (2015) [15]	400	Functional compromise reflected by sarcopenia, frailty, and nutritional depletion predicts adverse postoperative outcome after colorectal cancer surgery	Annals of Surgery	6.4	Q1
6	Robinson (2013) [16]	384	Simple frailty score predicts postoperative complications across surgical specialties	American Journal of Surgery	2.7	Q1
7	Carli (2020) [17]	353	Effect of multimodal prehabilitation vs postoperative rehabilitation on 30-day postoperative complications for frail patients undergoing resection of colorectal cancer: a randomized clinical trial	JAMA Surgery	14.9	Q1
8	Hall (2017a) [18]	343	Development and initial validation of the risk analysis index for measuring frailty in surgical populations	JAMA Surgery	14.9	Q1
9	Dasgupta (2009) [19]	338	Frailty is associated with postoperative complications in older adults with medical problems	Archives of Gerontology and Geriatrics	3.8	Q2
10	Kristjansson (2024) [20]	331	Comprehensive geriatric assessment can predict complications in elderly patients after elective surgery for colorectal cancer: A prospective observational cohort study	Critical Reviews in Oncology/Hematology	6.2	Q1

The paper “Frailty and Cancer: Implications for Oncology Surgery, Medical Oncology, and Radiation Oncology” was published in *CA: A Cancer Journal for Clinicians*, which has the highest impact factor of 232.4. The study suggests that continuous efforts by oncology professionals to identify frailty and promote multidisciplinary decision-making will aid in creating personalized management strategies, thereby improving cancer patient care.

Table 4 present a compilation of the top ten co-cited references within PFA for EP [11], [12], [21], [22], [23], [24], [25], [26], [27], [28]. The article entitled “Frailty as a Predictor of Surgical Outcomes in Older Patients” [11] and “Accumulating deficits model of frailty and postoperative mortality and morbidity: its application to a national database” [12], both articles ranked among the top 10 in terms of citation count and co-citation frequency. Fifty percent of these papers have been made available between 2012 and 2018, highlighting their significant additions to the field and continued influence. The 2001 article “Frailty in older adults: Evidence for a phenotype,” published in the *Journals of Gerontology Series A-Biological Sciences and Medical Sciences*, is one of the most frequently co-cited references, with over 500 co-citations. Therefore, the article entitled “Frailty in elderly people” [28], published in the *Lancet*, have the highest impact factor (IF = 88.5).

**Table 4:** The top 10 co-cited references related to research of preoperative frailty assessment for elderly patients.

Ranks	References	Co-citations	Title	Journal	IF(2024)	JCR
1	Fried (2001) [21]	566	Frailty in older adults: evidence for a phenotype	Journals of Gerontology Series A-Biological Sciences and Medical Sciences	3.8	Q1
2	Makary (2010) [11]	374	Frailty as a predictor of surgical outcomes in older patients	Journal of The American College of Surgeons	3.4	Q1
3	Rockwood (2005) [22]	343	A global clinical measure of fitness and frailty in elderly people	Canadian Medical Association Journal	11.3	Q1
4	Dindo (2004) [23]	206	Classification of surgical complications: a new proposal with evaluation in a cohort of 6336 patients and results of a survey	Annals of Surgery	6.4	Q1
5	Charlson (1987) [24]	203	The therapeutic efficacy of critical care units from two perspectives: a traditional cohort approach vs a new case-control methodology	Journal of Chronic Diseases	/	/
6	Lin (2016) [25]	185	Frailty and post-operative outcomes in older surgical patients: a systematic review	BMC Geriatrics	3.8	Q1
7	Velanovich (2013) [12]	179	Accumulating deficits model of frailty and postoperative mortality and morbidity: its application to a national database.	Journal of Surgical Research	1.7	Q2
8	Chow (2012) [26]	176	Relevance of the c-statistic when evaluating risk-adjustment models in surgery	Journal of The American College of Surgeons	3.4	Q1
9	Subramaniam (2018) [27]	174	New 5-Factor Modified Frailty Index Using American College of Surgeons NSQIP Data	Journal of The American College of Surgeons	3.4	Q1
10	Clegg (2013) [28]	162	Frailty in elderly people	Lancet	88.5	Q1

### 3.7 Keywords

Fig. 5A presents the 25 most-cited keywords. Notably, Among the top 25 keywords, “frailty,” “mortality,” “outcomes,” “surgery,” “complication” and “morbidity” are significant in the conversation of the aforementioned field, highlighting the key concepts of the research investigation.

**Fig. 5:**
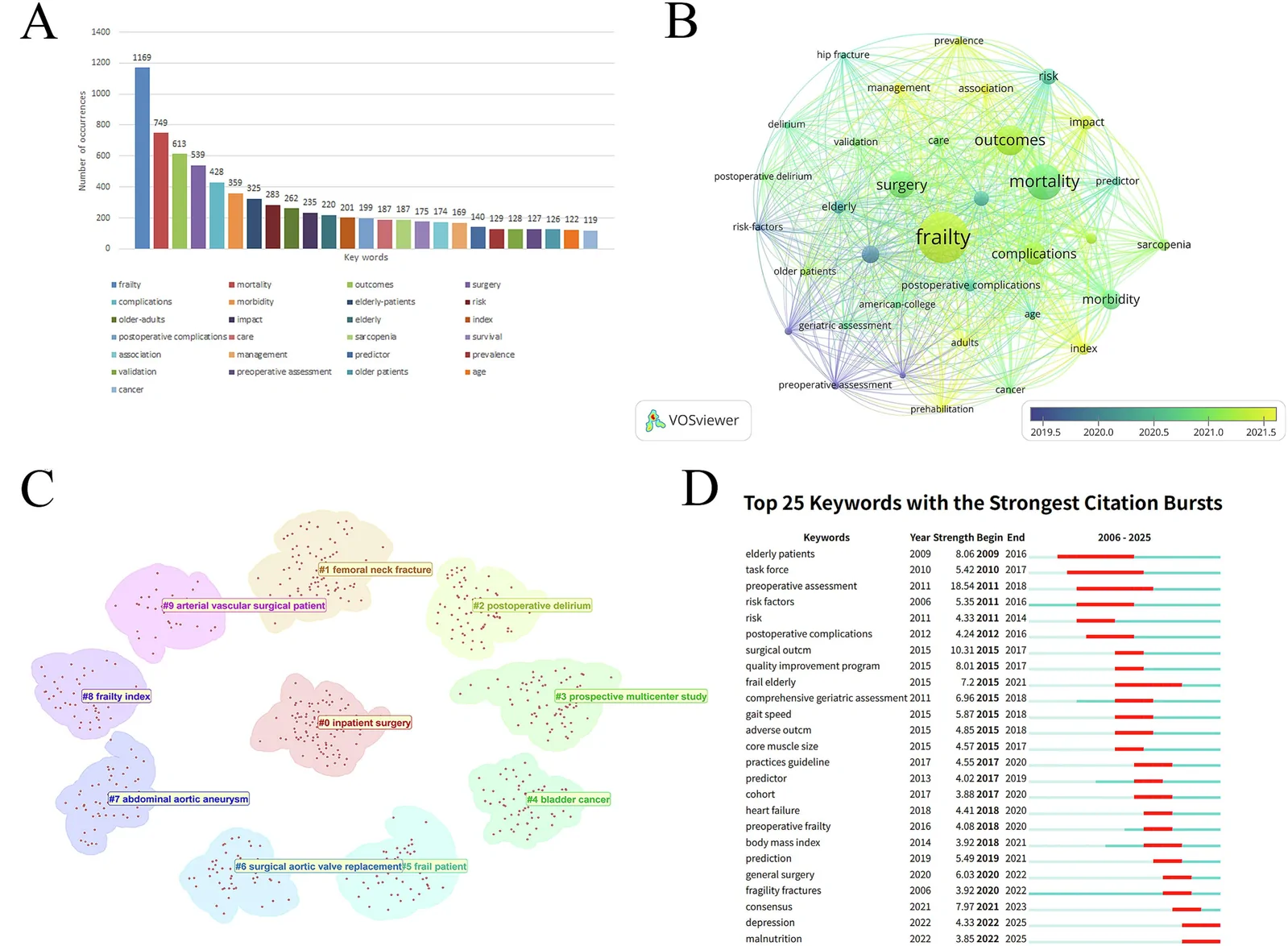
Visualization of keywords analysis in terms of research of preoperative frailty assessment for elderly patients. (A) The top 20 keywords in the field; (B)Time-overlapping network; (C)Cluster analysis of keywords; (D)The top 25 keywords with the strongest citation bursts.

Fig. 5B illustrates the rate of appearance and the temporal connection of connections among these keywords. Terms released in previous decades, such as “preoperative assessment,” “risk-factor,” and “elderly,” were highlighted in purple. In the last three years, researchers have increasingly concentrated on the concepts of “index,” “outcomes,” “sarcopenia,” and “management,” suggesting that these topics have been pivotal in recent studies and are likely to continue to be significant moving forward.

Fig. 5C illustrates clusters categorized according to how they relate to topic of theme: Cluster #0 pertains to inpatient surgery, Cluster #1 to femoral neck fracture, Cluster #2 to postoperative delirium, Cluster #3 to postoperative multicenter study, Cluster #4 to bladder cancer, Cluster #5 to frail patient, Cluster #6 to surgical aortic replacement, Cluster #7 to abdominal aortic aneurysm, Cluster #8 to frailty index, Cluster #9 to arterial vascular surgical patient.

Fig. 5D presents the top 25 keywords with the most significant citation bursts, thereby successfully illustrating the evolving trends and areas of focus within PFA for EP. Significantly, “preoperative assessment” exhibited the highest intensity of reference bursting, recorded at a value of 21.64. Additionally, the terms “surgical outcome,” “elderly patients,” “quality improvement program,” “consensus,” “frail elderly,” “comprehensive geriatric assessment,” “general surgery,” “gait speed,” and “prediction” exhibit a burst strength exceeding 5, signifying their significance and active research focus in PFA for EP. The line in red illustrates the duration of the citation explosion, whereas the blue line denotes the time interval. This map depicts the temporal evolution of research centers. The concepts of “depression” and “malnutrition” have received considerable focus in the past decade.

Based on the aforementioned bibliometric indicators, three specific emerging hotspots were identified for in-depth discussion. First, ‘sarcopenia’ was identified as a critical frontier topic; it occupies a prominent position in the recent time-overlapping network (Fig. 5B) and is a core research subject, as evidenced by two of the top 10 most cited documents explicitly featuring it in their titles (Table 3). Second, ‘depression’ and ‘malnutrition’ were selected because they exhibited distinct citation bursts during the 2022–2025 period (Fig. 5D), marking them as the immediate focal points of recent research. Therefore, these three domains represent the transition from macro-level frailty screening to micro-level targeted clinical interventions.

## 4 Discussions

### 4.1 General information

This is the first study to follow PFA research for EP from 2005 to 2025 using bibliometrics. Depending on the topic, bibliometrics analysis may summarize current knowledge and predict future development patterns, in contrast to systematic and scope-defining evaluation. We looked at national regions, research institutions, journals, authors, documents, keywords, and bibliometrics and visual analytic tools to summarize PFA for EP knowledge and find new topic research hotspots.

A growing tendency in publications is shown by the current analysis of PFA for EP research. PFA has been used by several medical facilities over the last 20 years due to its advantages of fewer problems and quicker recovery after surgery. While China's current expansion is in line with government-sponsored hospital equipment improvements, the US and Europe dominate PFA research because to their sophisticated surgical techniques, robust financing, and specialized facilities.

Establishing the most dependable large-scale PFA tests requires international cooperation across countries, organizations, and writers. One of the top surgical journals known for its depth and scope is *JAMA Surgery*, which has the highest IF and the most citations. It was not surprising that *JAMA Surgery* led PFA for EP research since PFA advances was often driven by clinical trials. The majority of the top 10 most cited articles and co-cited references are published in JCR Q1 journals, including prestigious journals with impact factors over 50. This indicates that research on PFA for EP has garnered substantial global attention and recognition. Additionally, for those seeking to rapidly comprehend the current research landscape of the field and forecast its future trajectories, the afore mentioned 18 articles are indispensable [11], [12], [13], [14], [15], [16], [17], [18], [19], [20], [21], [22], [23], [24], [25], [26], [27], [28].

The bibliometric data provide clear evidence for the shifting research paradigms. As PFA evolved from macro-level screening to micro-level targeted management, specific clinical targets emerged as new focal points. ‘Sarcopenia’ has become a frontier research focus over the past three years (Fig. 5B) and a core subject in high-impact literature (Table 3). Concurrently, the keyword burst analysis (Fig. 5D) reveals that research focus shifted towards ‘depression’ and ‘malnutrition’ during the 2022–2025 period. Consequently, these three domains were identified as the most critical emerging hotspots for further clinical discussion.

### 4.2 Sarcopenia

Sarcopenia is an age-related disease characterized by accelerated loss of muscle mass and function, which not only impairs physical performance and mobility in older adults but also elevates the risk of falls, fractures, and premature mortality [29], [30]. A study of 318 elderly patients with coronary heart disease revealed a sarcopenia prevalence of 21.70%, which was significantly associated with reduced cardiac output, lower left ventricular ejection fraction, and higher mortality [31]. Furthermore, sarcopenic patients undergoing cardiac surgery experienced markedly prolonged hospital stays, exacerbating healthcare burdens [32].

PFA plays a pivotal role in managing sarcopenia in elderly patients by providing a multidimensional evaluation encompassing physical, functional, cognitive, psychological, and social domains. This holistic approach enables personalized care plans tailored to individual needs. Studies demonstrate that PFA enhances diagnostic accuracy, optimizes patient outcomes, and reduces healthcare costs. Early identification and intervention—integrating nutritional support, resistance training, and targeted pharmacotherapy—can mitigate the progression of sarcopenia and its associated complications [33].

CT-based imaging assessment further demonstrates an independent association between sarcopenia and postoperative complications. In a study of elderly patients with femoral neck fractures, CT-quantified sarcopenia was significantly correlated with increased postoperative transfusion requirements. These findings underscore the clinical imperative of preoperative sarcopenia screening, which enables targeted interventions to mitigate adverse outcomes [34].

### 4.3 Depression

Depression is a prevalent mental health disorder in elderly surgical patients, exerting significant impacts on postoperative recovery and overall outcomes. Studies demonstrate a robust association between preoperative depression and increased postoperative complications, particularly in cardiovascular and major surgeries. Beyond exacerbating psychological distress, depression may dysregulate physiological homeostasis and immune function, thereby elevating risks of surgical complications such as delirium, prolonged hospitalization, and delayed functional recovery [35].

Depression is significantly more prevalent in cardiac patients compared to the general population, and depressive symptoms exacerbate cardiovascular risk factors while reducing adherence to evidence-based treatments and healthy lifestyle modifications [36]. Furthermore, preoperative depressive symptoms demonstrate a robust association with postoperative delirium, particularly in elderly patients, underscoring the critical need for preoperative mental health screening to mitigate adverse outcomes [37].

Depression has been identified as an independent predictor of poor postoperative recovery across diverse surgical types, including epilepsy surgery and major abdominal procedures. Studies demonstrate that preoperative depressive symptoms are significantly associated with prolonged hospital stays, which not only escalates healthcare resource utilization but may also impede long-term functional recovery [38]. Furthermore, depression correlates with increased risks of postoperative infections, hospital readmissions, and emergency department visits, underscoring its multifaceted impact on surgical outcomes. These findings highlight the critical need for integrating mental health screening into preoperative assessments to mitigate adverse postoperative sequelae [39].

Standardized screening pathways for early depression identification and management can significantly improve patient outcomes [40]. The CGA provides a holistic evaluation of elderly patients, encompassing depression, cognitive impairment, social status, multimorbidity, frailty, geriatric syndromes, nutritional status, and polypharmacy [41]. This integrated approach not only facilitates the identification of postoperative complication risks but also enables the development of personalized preoperative optimization strategies tailored to the unique needs of geriatric populations.

### 4.4 Malnutrition

Malnutrition is a critical component of preoperative frailty assessment, which has garnered significant attention for its impact on postoperative complications. A comparative study evaluating two malnutrition screening tools (PONS and NRS 2002) in elderly surgical patients demonstrated that malnutrition identified by PONS was significantly associated with an elevated risk of postoperative complications [42]. Further supporting this, a study utilizing the MUST (Malnutrition Universal Screening Tool) revealed that patients with high nutritional risk exhibited markedly increased postoperative mortality (OR: 11.9, *p* = 0.035) and prolonged hospital stays [43]. These findings underscore the pivotal role of malnutrition in preoperative frailty assessment, as poor nutritional status may exacerbate postoperative recovery challenges and complication rates through mechanisms such as impaired wound healing, immune dysfunction, and reduced physiological reserve.

Preoperative malnutrition has been consistently linked to increased postoperative mortality and complication risks in colorectal cancer patients, with hypoalbuminemia (serum albumin <3.5 g/dL) identified as a critical predictor of adverse outcomes [44]. Notably, hypoalbuminemia is prevalent among elderly surgical patients and independently associated with prolonged hospitalization and elevated mortality [45]. The Geriatric Nutritional Risk Index (GNRI) further validates this relationship, as patients with low GNRI scores exhibit significantly higher postoperative complication rates, particularly in elderly oncology cohorts [46]. These findings underscore the imperative of integrating nutritional screening and frailty assessment into standard preoperative protocols for geriatric patients, offering clinicians actionable insights to optimize perioperative management and improve surgical outcomes [4].

### 4.5 Limitations

It should be acknowledged that this research has number of intrinsic limitations. The literature review was restricted to the Web of Science Core Collection database, potentially excluding relevant studies. The selection process may have been biased due to the restriction of inclusion criteria to English-language publications. Finally, while the search strategy was highly specific to the preoperative surgical setting and restricted to articles and reviews, we did not perform a manual full-text relevance screening for all retrieved records. This might have included a few marginally relevant studies, which is a limitation inherent to large-scale bibliometric analyses.

## 5 Conclusions

This bibliometric analysis systematically evaluates global research trends in PFA for EP from 2005 to 2025, revealing exponential growth in publications and shifting research paradigms. Future research should prioritize large-scale, multidisciplinary studies to integrate emerging sarcopenia metrics, depression, and nutritional status into unified preoperative frameworks. Additionally, expanding collaboration between high- and low-resource regions could address disparities in frailty assessment adoption and refine risk stratification models.

## Assistance with the study

None.

## CRediT authorship contribution statement

**Yuling Ni:** Conceptualization, Data curation, Formal analysis, Visualization, Writing – original draft, Writing – review & editing. **Naiyang Tang:** Conceptualization, Investigation, Methodology, Project administration, Resources, Software, Supervision. **Xi Xu:** Conceptualization, Data curation, Supervision, Validation, Writing – original draft, Writing – review & editing.

## Consent for publication

This article does not contain any studies with human participants or animals performed by any of the authors.

## Ethics approval and consent to participate

This article does not contain any studies with human participants or animals performed by any of the authors.

## Declaration of generative AI and AI-assisted technologies in the writing process

During the preparation of this work the authors used DeepSeek in order to improve readability and language. After using this tool, the authors reviewed and edited the content as needed and take full responsibility for the content of the published article.

## Funding statement

This research did not receive any specific grant from funding agencies in the public, commercial, or not-for-profit sectors.

## Declaration of competing interest

The authors declare that they have no known competing financial interests or personal relationships that could have appeared to influence the work reported in this paper.

## Data Availability

The raw data supporting the conclusions of this article will be made available by the authors, without undue reservation.

## References

[1] Deng Y., Liao R., Hu X., Zhang K., Zhu J., Sato N. (2024 Dec 31). Prevalence of physical frailty and its associated factors among elderly patients undergoing hepatobiliary pancreatic surgery in China. Glob Health Med.

[2] George E.L., Hall D.E., Youk A., Chen R., Kashikar A., Trickey A.W. (2021 Jan 1). Association between patient frailty and postoperative mortality across multiple noncardiac surgical specialties. JAMA Surg.

[3] Ma W., Shui W., Peng Q., Zhu C., Zhao W., Fan G. (2025 Feb 19). Impact of preoperative frailty on new disability or death after cardiac surgery in elderly patients: a prospective cohort study. Front Med (Lausanne).

[4] Contreras V., Elgueta M.F., Balde D., Astaburuaga P., Carrasco M., Pedemonte J.C. (2024 Dec). Prehabilitation for Chilean frail elderly people - pre-surgical conditioning protocol - to reduce the length of stay: randomized control trial. Minerva Anestesiol.

[5] Di Donato V., Caruso G., Bogani G., Giannini A., D’Oria O., Perniola G. (2021 Apr). Preoperative frailty assessment in patients undergoing gynecologic oncology surgery: a systematic review. Gynecol Oncol.

[6] Kim D.H., Afilalo J., Shi S.M., Popma J.J., Khabbaz K.R., Laham R.J. (2019 Mar 1). Evaluation of changes in functional status in the year after aortic valve replacement. JAMA Intern Med.

[7] Shahrokni A., Tin A., Alexander K., Sarraf S., Afonso A., Filippova O. (2019 May 3). Development and evaluation of a new frailty index for older surgical patients with cancer. JAMA Netw Open.

[8] Xu X., Xu J., Gao H., Sheng Z., Xu Y., Zeng S. (2025 Apr). Bibliometric analysis of photodynamic research in bladder cancer: trends and future directions. Photodiagnosis Photodyn Ther.

[9] Li C., Cheng Y., Li Z., Margaryan D., Perka C., Trampuz A. (2021 Feb 17). The pertinent literature of enhanced recovery after surgery programs: a bibliometric approach. Medicina (Kaunas).

[10] Chen Q., Wang Y., Song S., Xie G., Cheng B., Fang X. (2022 Apr). Global research trends in perioperative care for the elderly: a bibliometric and visualized study. J Anesth.

[11] Makary M.A., Segev D.L., Pronovost P.J., Syin D., Bandeen-Roche K., Patel P. (2010 Jun). Frailty as a predictor of surgical outcomes in older patients. J Am Coll Surg.

[12] Velanovich V., Antoine H., Swartz A., Peters D., Rubinfeld I. (2013 Jul). Accumulating deficits model of frailty and postoperative mortality and morbidity: its application to a national database. J Surg Res.

[13] Ethun C.G., Bilen M.A., Jani A.B., Maithel S.K., Ogan K., Master V.A. (2017 Sep). Frailty and cancer: implications for oncology surgery, medical oncology, and radiation oncology. CA Cancer J Clin.

[14] Peng P., Hyder O., Firoozmand A., Kneuertz P., Schulick R.D., Huang D. (2012 Aug). Impact of sarcopenia on outcomes following resection of pancreatic adenocarcinoma. J Gastrointest Surg.

[15] Reisinger K.W., van Vugt J.L., Tegels J.J., Snijders C., Hulsewé K.W., Hoofwijk A.G. (2015 Feb). Functional compromise reflected by sarcopenia, frailty, and nutritional depletion predicts adverse postoperative outcome after colorectal cancer surgery. Ann Surg.

[16] Robinson T.N., Wu D.S., Pointer L., Dunn C.L., Cleveland J.C., Moss M. (2013 Oct). Simple frailty score predicts postoperative complications across surgical specialties. Am J Surg.

[17] Carli F., Bousquet-Dion G., Awasthi R., Elsherbini N., Liberman S., Boutros M. (2020 Mar 1). Effect of multimodal prehabilitation vs postoperative rehabilitation on 30-day postoperative complications for frail patients undergoing resection of colorectal cancer: a randomized clinical trial. JAMA Surg.

[18] Hall D.E., Arya S., Schmid K.K., Blaser C., Carlson M.A., Bailey T.L. (2017 Feb 1). Development and initial validation of the risk analysis index for measuring frailty in surgical populations. JAMA Surg.

[19] Dasgupta M., Rolfson D.B., Stolee P., Borrie M.J., Speechley M. (2009). Frailty is associated with postoperative complications in older adults with medical problems. Arch Gerontol Geriatr.

[20] Kristjansson S.R., Nesbakken A., Jordhøy M.S., Skovlund E., Audisio R.A., Johannessen H.O. (2010 Dec). Comprehensive geriatric assessment can predict complications in elderly patients after elective surgery for colorectal cancer: a prospective observational cohort study. Crit Rev Oncol Hematol.

[21] Fried L.P., Tangen C.M., Walston J., Newman A.B., Hirsch C., Gottdiener J. (2001 Mar). Frailty in older adults: evidence for a phenotype. J Gerontol A Biol Sci Med Sci.

[22] Rockwood K., Song X., MacKnight C., Bergman H., Hogan D.B., McDowell I. (2005 Aug 30). A global clinical measure of fitness and frailty in elderly people. CMAJ.

[23] Dindo D., Demartines N., Clavien P.A. (2004 Aug). Classification of surgical complications: a new proposal with evaluation in a cohort of 6336 patients and results of a survey. Ann Surg.

[24] Charlson M.E., Sax F.L. (1987). The therapeutic efficacy of critical care units from two perspectives: a traditional cohort approach vs a new case-control methodology. J Chronic Dis.

[25] Lin H.S., Watts J.N., Peel N.M., Hubbard R.E. (2016 Aug 31). Frailty and post-operative outcomes in older surgical patients: a systematic review. BMC Geriatr.

[26] Merkow R.P., Hall B.L., Cohen M.E., Dimick J.B., Wang E., Chow W.B. (2012 May). Relevance of the c-statistic when evaluating risk-adjustment models in surgery. J Am Coll Surg.

[27] Subramaniam S., Aalberg J.J., Soriano R.P., Divino C.M. (2018 Feb). New 5-factor modified frailty index using American college of surgeons NSQIP data. J Am Coll Surg.

[28] Clegg A., Young J., Iliffe S., Olde M., Rockwood K. (2013). Frailty in elderly people. Lancet.

[29] Wang Y., Meilu D., Qi S., Yufei T., Song G., Susu H. (2025 Jul 16). The impact of sarcopenia on prognosis and fruquintinib efficacy in advanced colorectal cancer: a retrospective and mendelian randomization study. Front Immunol.

[30] Sayer A.A., Cooper R., Arai H., Cawthon P.M., Ntsama Essomba M.J., Fielding R.A. (2024 Sep 19). Sarcopenia. Nat Rev Dis Primers.

[31] Li H., Ma F., Li Y., Li H., Huang J. (2025 Jan 17). Occurrence of sarcopenia in elderly patients with coronary heart disease and its association with short-term prognosis. BMC Cardiovasc Disord.

[32] Won M.H., Yun K.H., Kim H., Son Y.J. (2024 Apr 12). Prognostic role of sarcopenia on major adverse cardiac events among patients who underwent successful percutaneous coronary intervention: a retrospective cohort study. Eur J Cardiovasc Nurs.

[33] Jia Q., Pan Y., Pan D., Xia Y., Wu J. (2025 Aug 26). Application of comprehensive geriatric assessment (CGA) in assessment, management and prognosis of older adults patients with sarcopenia: a review of research progress and practice. Gerontology.

[34] Li L., Yang H., Zhang Y., Ren Z., Lin Z., Li Z. (2025 Aug 20). CT-based sarcopenia is independently associated with blood transfusions after hip arthroplasty in elderly patients with femoral neck fractures: a retrospective cohort study. BMC Geriatr.

[35] Tigges-Limmer K., Sitzer M., Gummert J. (2021 May 17). Perioperative psychological interventions in heart surgery–opportunities and clinical benefit. Dtsch Arztebl Int.

[36] Jha M.K., Qamar A., Vaduganathan M., Charney D.S., Murrough J.W. (2019 Apr 16). Screening and management of depression in patients with cardiovascular disease: JACC state-of-the-art review. J Am Coll Cardiol.

[37] Smith P.J., Attix D.K., Weldon B.C., Monk T.G. (2016 Mar). Depressive symptoms and risk of postoperative delirium. Am J Geriatr Psychiatry.

[38] Orri M., Boleslawski E., Regimbeau J.M., Barry C., Hassler C., Gregoire E. (2015 Nov). Influence of depression on recovery after major noncardiac surgery: a prospective cohort study. Ann Surg.

[39] Xu E., Karsalia R., Arena J., Gor A., Malhotra R.D., Kost J. (2026). Positive preprocedure depression screening is associated with worse outcomes after single-level posterior-only lumbar fusion: a retrospective cohort analysis. Neurosurgery.

[40] Hietamies T.M., Smith A.E., Lii T.R., Muzzall E., Flohr J., Okada R.L. (2024 Jul). Prevalence and characteristics of preoperative patients with depression. Br J Anaesth.

[41] Schipa C., Luca E., Ripa M., Sollazzi L., Aceto P. (2023). Preoperative evaluation of the elderly patient. Saudi J Anaesth.

[42] Zhang F., He S.T., Zhang Y., Mu D.L., Wang D.X. (2021 Jun 21). Comparison of two malnutrition assessment scales in predicting postoperative complications in elderly patients undergoing noncardiac surgery. Front Public Health.

[43] Venianaki M., Andreou A., Nikolouzakis T.K., Chrysos E., Chalkiadakis G., Lasithiotakis K. (2021 Jun 9). Factors associated with malnutrition and its impact on postoperative outcomes in older patients. J Clin Med.

[44] Hu W.H., Cajas-Monson L.C., Eisenstein S., Parry L., Cosman B., Ramamoorthy S. (2015 Sep 7). Preoperative malnutrition assessments as predictors of postoperative mortality and morbidity in colorectal cancer: an analysis of ACS-NSQIP. Nutr J.

[45] Tischler E.H., McDermott J.R., Vummidi S., Mahmoud S.A., Gross J.M., Malik A.N. (2024 Dec 18). Is it time to reassess the role of preoperative hypoalbuminemiaamong geriatric distal femur fracture patients?. Arch Orthop Trauma Surg.

[46] Fukuta K., Daizumoto K., Sasaki Y., Izumi K., Kadoriku F., Utsunomiya S. (2025 Aug). Geriatric nutritional risk index predicts postoperative complications in elderly patients undergoing robot-assisted radical cystectomy. Int J Urol.

